# LRAT coordinates the negative-feedback regulation of intestinal retinoid biosynthesis from β-carotene

**DOI:** 10.1016/j.jlr.2021.100055

**Published:** 2021-02-23

**Authors:** Srinivasagan Ramkumar, Jean Moon, Marcin Golczak, Johannes von Lintig

**Affiliations:** 1 Department of Pharmacology, School of Medicine, Case Western Reserve University, Cleveland, OH, USA; 2 Cleveland Center for Membrane and Structural Biology, School of Medicine, Case Western Reserve University, Cleveland, OH, USA

**Keywords:** enterocytes, retinoids, carotenoids, cell signaling, metabolism, lipids, LRAT, vitamin A, BC, β-carotene, BCO1, β-carotene oxygenase-1, CCR9, C-C motif chemokine receptor 9, CD36, cluster of differentiation 36, DKO, double knockout, dRA, deuterated retinoic acid, ISX, intestine-specific homeodomain transcription factor, KLF4, krüppel-like factor 4, LRAT, lecithin:retinol acyltransferase, RA, retinoic acid, RARs, RA receptors, REs, retinyl esters, ROL, retinol, SR-B1, scavenger receptor class B type 1, VAD, vitamin A deficiency

## Abstract

There is increasing recognition that dietary lipids can affect the expression of genes encoding their metabolizing enzymes, transporters, and binding proteins. This mechanism plays a pivotal role in controlling tissue homeostasis of these compounds and avoiding diseases. The regulation of retinoid biosynthesis from β-carotene (BC) is a classic example for such an interaction. The intestine-specific homeodomain transcription factor (ISX) controls the activity of the vitamin A-forming enzyme β-carotene oxygenase-1 in intestinal enterocytes in response to increasing concentration of the vitamin A metabolite retinoic acid. However, it is unclear how cells control the concentration of the signaling molecule in this negative-feedback loop. We demonstrate in mice that the sequestration of retinyl esters by the enzyme lecithin:retinol acyltransferase (LRAT) is central for this process. Using genetic and pharmacological approaches in mice, we observed that in LRAT deficiency, the transcription factor ISX became hypersensitive to dietary vitamin A and suppressed retinoid biosynthesis. The dysregulation of the pathway resulted in BC accumulation and vitamin A deficiency of extrahepatic tissues. Pharmacological inhibition of retinoid signaling and genetic depletion of the *Isx* gene restored retinoid biosynthesis in enterocytes. We provide evidence that the catalytic activity of LRAT coordinates the negative-feedback regulation of intestinal retinoid biosynthesis and maintains optimal retinoid levels in the body.

Vitamin A plays critical roles in mammalian biology as precursor for at least two critical compounds. The vitamin A metabolite retinoic acid (RA) is a small molecule regulator that elicits profound cellular responses through retinoic acid receptors (RARs) (1, 2). These transcription factors form obligate dimers with retinoid X receptors (3) and control the transcription of about 500 target genes in the human genome (4, 5). The vitamin A metabolite retinal exists in significant concentrations in photoreceptors of the retina and serves as the chromophore of visual pigments (6). These G-protein-coupled receptors mediate phototransduction, the process that converts light into a nervous signal (7).

To maintain these functions, the body acquires vitamin A precursors from the diet, enzymatically metabolizes, and distributes them through the circulation (8). These precursors exist either as provitamin A, mainly β-carotene (BC), or as preformed vitamin A, mainly retinyl esters (REs). Excessive intake of preformed vitamin A is toxic (hypervitaminosis A). Symptoms include impaired vision, vomiting, bone pain or swelling, skin problems, headache, liver damage, and nausea (9). Excessive intake of BC does not cause hypervitaminosis A but carotenemia (BC accumulation) (10), as its conversion to retinoids by the enzyme β-carotene oxygenase-1 (BCO1) is regulated by enterocytes of the intestine to maintain an optimum level of vitamin A (11).

Initial biochemical studies showed that the vitamin A status of the host regulates BCO1 enzyme activity (12, 13). After cloning of the *BCO1* gene, evidence was provided that the regulation of BCO1 occurs at the transcriptional level and involves RAR signaling (14). Later studies showed that RARs control the expression of the intestine-specific homeodomain transcription factor (ISX) (15). In response to retinoid signaling (16), the transcription factor suppresses the expression of the gene encoding scavenger receptor class B type 1 (SR-B1) (*Scarb1*) and of *Bco1* gene (17). Accordingly, ISX-deficient mice display increased absorption of carotenoids (18) and carotenoid conversion to retinoids (19).

Though much progress has been made in identifying the molecular components in this negative-feedback loop, it is unclear how the enterocytes adjust the RA concentration and control ISX activity. A premature shutoff of *Bco1* expression by ISX would put the organism at risk of systemic vitamin A deficiency (VAD), and an uncontrolled BCO1 activity would result in hypervitaminosis A. Both scenarios are detrimental and can impair vitamin A-dependent processes in vision, embryonic development, immunity, and metabolism (8, 20, 21, 22).

We previously observed in cells and tissues that RA concentrations increase with impaired lecithin:retinol acyltransferase (LRAT) function (23, 24). LRAT acts downstream in the pathway for retinoid biosynthesis and esterifies retinol (ROL) by transferring an acyl moiety from the sn-1 position of phosphatidylcholine (25, 26). REs are the transport form of vitamin A in triglyceride-rich lipoproteins and the storage form of vitamin A in the liver and other tissues (27). However, the putative role of LRAT in the control of retinoid biosynthesis from BC has not been established.

We here hypothesized that LRAT activity is critical for the control of retinoid biosynthesis in enterocytes of the intestine and optimal vitamin A levels in the body. Thus, we exploited *Lrat*^*−/−*^ (28) and *Isx*^*−/−*^ mice (15) as well as RAR antagonists to elucidate the interplay of the different components in the negative-feedback loop that controls retinoid biosynthesis.

## Materials and methods

### Animal care and husbandry

Case Western Reserve University Animal Care and Use Committee approved the procedures for the mouse experiments. The generation of *Lrat*^*−/−*^ and *Isx*^*−/−*^ mice has been previously described (15, 28). For the generation of *Isx*^*−/−*^*/Lrat*^*−/−*^ double knockout (DKO) mice, we crossed *Isx*^*−/−*^ mice with *Lrat*^*−/−*^ mice. We purchased WT mice from Jackson Laboratories (Bar Harbor, ME). All mice were on a B6 (Cg)-Tyrc-2j/j genetic background. We maintained mice at 24°C in a 12/12 h light-dark cycle, and animals had free access to food and water. We used female mice in all experiments to reduce gender-specific variability. During the breeding and weaning periods, we maintained mice on standard rodent diet containing ∼15,000 IU vitamin A/kg diet (Laboratory rodent diet 5001; LabDiet, St. Louis, MO). For the short-term experiment, we kept freshly weaned WT and *Lrat*^*−*/−^ mice on an American Institute of Nutrition rodent diet (20% protein, 64% carbohydrate, and 7% fat) without added vitamin A for 7 days. Then we fed mice with the same diet supplemented with vitamin A (15,000 IU vitamin A, retinyl acetate) or BC (25 mg/kg). The diets were prepared by Research Diets, Inc. (New Brunswick, NJ). BC was incorporated as water-soluble beadlets (DSM Ltd., Sisseln, Switzerland). For the long-term intervention, WT, *Lrat*^*−/−*^, and DKO mice were kept on vitamin A-deficient diet for 1 week and then transferred to the same diet (VAD group) or transferred to diets containing vitamin A (15,000 IU vitamin A) or BC (25 mg/kg). After indicated time points, mice were anesthetized by intraperitoneal injection of rodent cocktail mixture (ketamine 20 mg/ml with xylazine 1.7 mg/ml). Blood was drawn directly via cardiac puncture. Mice were perfused with 20–30 ml of sterile PBS and then sacrificed by cervical dislocation. Small intestine, liver, and gonadal white fat were dissected. Using the stomach as landmark, we defined the first third of the small intestine as duodenum, the second third as jejunum, and the third as ileum. After dissection, tissues were snap frozen in liquid nitrogen and stored in −80°C until further use.

For RAR antagonist treatment, freshly weaned *Lrat*^*−/−*^ (*n* = 6) were fed with BC diet (25 mg/kg; Research Diets, Inc.) for 1 week. After 1 week of dietary intervention, mice were separated into two groups with *n* = 3 for each group. One group of mice was gavaged with pan-RAR antagonist AGN193109 (Tocris Biosciences, Bristol, UK) at the concentration of 5 mg/kg body weight in sesame oil. The other group of mice was gavaged with sesame oil vehicle alone. The gavage was repeated 24 h later, and 6 h post the second gavage, mice were anesthetized by intraperitoneal injection of a rodent cocktail mixture containing 20 mg of ketamine, 7.5 mg of xylazine, with a dose of 0.2 ml per 25 g of mouse. Mice were then perfused with 20 ml of PBS and sacrificed by cervical dislocation.

### Isolation of intestinal crypts and villi

The entire small intestine was dissected from mice and used for isolation of villi and crypts. Briefly, small intestine was transferred into ice-cold PBS. Then, the intestine was flushed with ice-cold PBS to remove fecal matters without rupturing the tissue. The washed intestine was opened longitudinally with scissors and cut into three to five fragments. Each fragment was washed to remove any residual mucus and transferred into ice-cold PBS. The procedure was repeated several times until all mucus was removed (clear PBS supernatant). After the wash, tissues were transferred into fresh ice-cold PBS containing 5 mM EDTA and incubated at 4°C for 1 h with gentle shaking. After incubation, the tissues were laid on a glass slab on ice. A glass slide was used to gently remove villi from the intestine. The intestine pieces were incubated in ice-cold PBS containing 5 mM EDTA for 10 min at 4°C. After incubation, the samples were vigorously agitated to release the crypts. The supernatant was passed through a 70 μm filter. The filtered fraction contained some villi and mostly crypts. Filtration was repeated until the supernatant contained almost pure crypts. The isolation of villi and crypts was confirmed using light microscope at 8× magnification.

### HPLC analysis

Retinoids and carotenoids were extracted from 10 mg of liver, 50 mg of jejunum, and gonadal white adipose tissues. Adipose tissue samples were saponified prior to lipid extraction as previously described (29). For quantification of BC and retinoids, tissues were homogenized in a glass potter with 200 μl of PBS, and retinoids and BC were extracted with a mixture containing 200 μl of methanol and 400 μl of acetone and 500 μl of hexanes. HPLC analysis was carried out with an Agilent 1260 Infinity Quaternary HPLC system (Santa Clara, CA) equipped with a pump (G1312C) with an integrated degasser (G1322A), a thermostated column compartment (G1316A), an autosampler (G1329B), a diode-array detector (G1315D), and online analysis software (ChemStation, Agilent Technologies). The analyses were carried out at 25°C using a normal-phase Zorbax Sil (5 μm, 4.6 × 150 mm) column (Agilent Technologies, Santa Clara, CA) protected with a guard column with the same stationary phase. Chromatographic separation was achieved by isocratic flow of 10% ethyl acetate/90% hexanes. For quantification of molar amounts of retinoids and BC, the HPLC has previously been scaled with synthesized standard compounds.

### RA extraction and LC/MS analysis

For RA extraction, 100 mg of liver and jejunum samples were homogenized in 1 ml of PBS (pH 7.4) and transferred to disposable glass culture tubes (16 × 150 mm). A volume of 1–3 ml of freshly prepared 0.025 M KOH in ethanol was added, and samples were vortexed. About 1 ml of acetonitrile was added and vortexed. A volume of 10–15 ml of hexanes was added, and the mixture was vortexed. Phase separation was achieved by centrifugation at 500 *g*. The organic top layer was removed and contained no polar retinoids. To acidify the remaining ethanolic layer, 50–100 μl of 6 M HCl were added, and the mixture was vortexed. Then, 10 ml of hexanes were added, and the mixture was vortexed. Phase separation was achieved by centrifugation at 500 *g* for 3 min. The organic phase was removed with a glass pipette and transferred in a new glass vial. The organic solvent was evaporated under a constant stream of nitrogen. The debris was dissolved in 100–200 μl acetonitrile containing 0.1% formic acid.

RA was separated on the Hypersill Gold 50 × 2.1 column (Thermo Fisher Scientific) by a linear gradient of water → acetonitrile (50% → 100% in 5 min followed by 100% acetonitrile for 10 min) at a flow rate of 0.3 ml/min. Both solvents contained 0.1% formic acid. MS-based detection and quantification of RA was performed with a linear trap quadrupole ion trap mass spectrometer (Thermo Fisher Scientific, Foster City, CA) equipped with an electrospray ionization interface operated in the positive ionization mode. The MS parameters were optimized for RA synthetic standard (Cayman Chemicals, Ann Arbor, MI). The quantification of endogenous RA was based on the known amount of deuterated RA (dRA) (Cayman Chemicals) that served as an internal standard added to the samples prior to tissue extraction and LC/MS analysis. RA and dRA were detected in the selected reaction monitoring mode using the following ion transition: 301.2 → 201.2 and 306.3 → 206.2 for RA and dRA, respectively. The relationship between the selected reaction monitoring ion intensity peaks was used to calculate the amount of endogenous RA. The experiment was performed in triplicates and repeated twice.

### RNA extraction and quantitative RT-PCR analysis

Liver and jejunum samples were collected (*n* = 4–5 per intervention group and genotype) and stored at −80°C until use. RNA was extracted from liver and jejunum by TRIzol method (Invitrogen, Carlsbad, CA). Total RNA was quantified using NanoDrop ND-1000 spectrophotometer (Thermo Fisher Scientific, Waltham, MA). cDNA was generated using the High Capacity RNA to cDNA kit (Applied Biosystems; Thermo Fisher Scientific). Gene expression measurement was carried out by real-time quantitative PCR using Applied Biosystems Real-Time PCR Instrument with TaqMan probes (Applied Biosystems; Thermo Fisher Scientific) primer β-actin (Mm02619580), *Isx* (Mm01243743), *Scarb1* (Mm00450234), *Bco1* (Mm01251350), *Aldh1a1* (Mm00657317), *cluster of differentiation 36* (*Cd36*) (Mm00432403), *Cyp26a1* (Mm00514486), *Rarb* (Mm01319677), krüppel-like factor 4 (*Klf4*) (Mm00516104), C-C motif chemokine receptor 9 (*Ccr9*) (Mm02620030), and *Muc2* (Mm01276696). Amplification was carried out using TaqMan Fast Universal PCR Master Mix (2X), no AmpErase UNG (Applied Biosystems; Thermo Fisher Scientific, Foster city, CA) following manufacturer's protocol. About 10 ng of cDNA was used per 10 μl reaction mixture. Gene expression levels were normalized to expression using a ΔΔC_t_ method.

### Statistical analysis

Data shown are the means ± SD. Analysis was performed using unpaired two-tailed *t*-test and one-way ANOVA using GraphPad Prism 8.0 software (GraphPad Software), and results were considered significant at ∗*P* < 0.05, ∗∗*P* < 0.005, and ∗∗∗*P* < 0.0001.

## Results

### Comparison of retinoid biosynthesis in WT and LRAT-deficient mice

**Figure 1:**
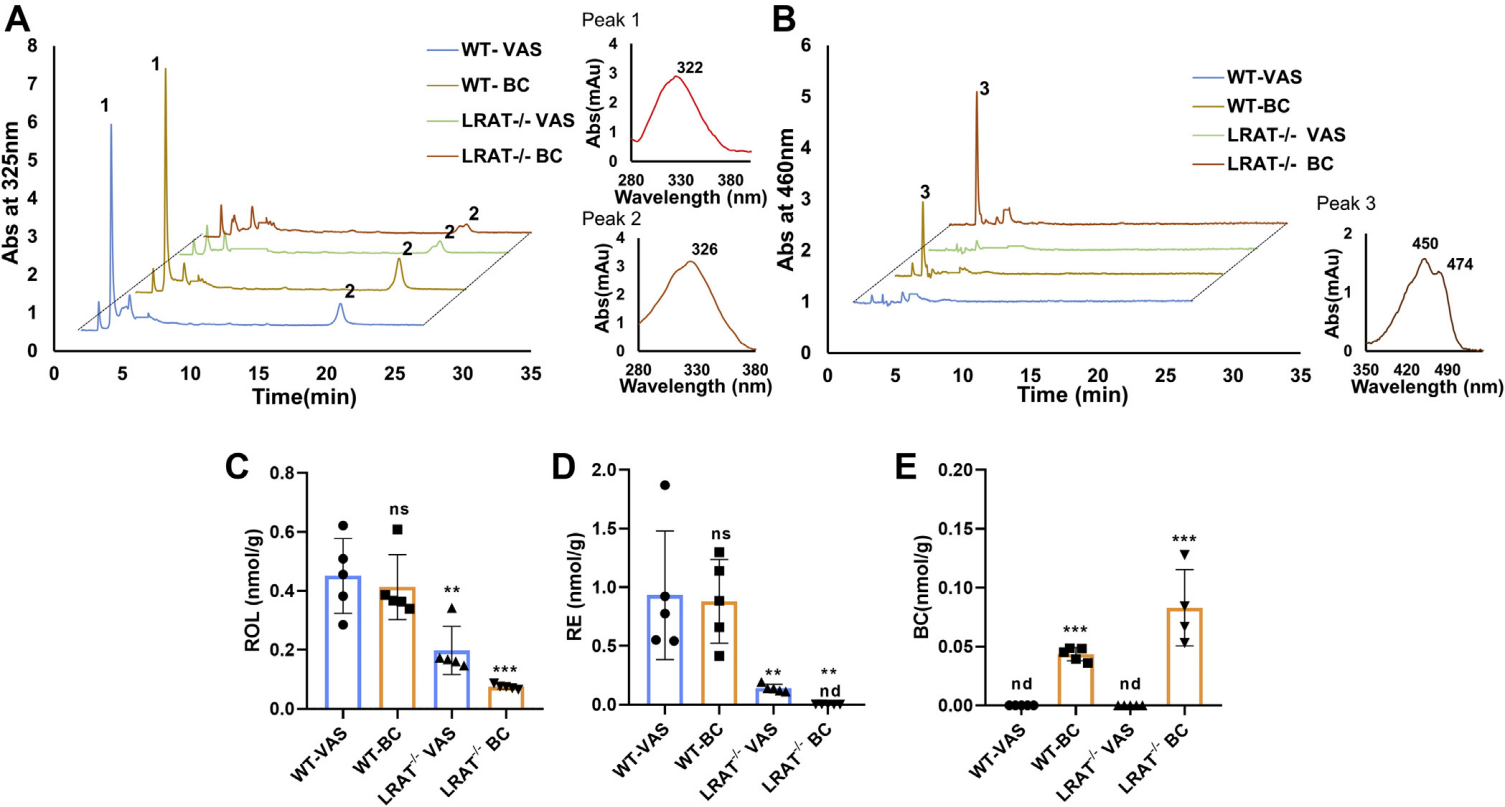
Retinoid concentrations in the jejunum of *Lrat*^*−/−*^ and WT mice on different diets. A: Representative HPLC chromatograms at 325 nm of lipid extracts of the jejunum of a WT and *Lrat*^*−/−*^ mice fed with diets supplemented with vitamin A [vitamin A sufficiency (VAS)] and β-carotene (BC). Peak 1 and 2 represents retinyl esters (REs) and all-*trans*-retinol (ROL), respectively. The spectral characteristics of peaks 1 and 2 are shown on the right. B: Representative HPLC chromatograms of the same lipid extracts at 460 nm. Peak 3 represents BC. The spectral characteristics of peak 3 are shown on the right. C: Quantification of ROL. D: Quantification of RE. E: Quantification of BC. The values in panels (C–E) represent means ± SD of data from at least five animals per genotype and diet. ∗*P* < 0.05, ∗∗*P* < 0.005, and ∗∗∗*P* < 0.0001. The statistical analysis was performed using one-way ANOVA by comparing WT-VAS as control. nd, not detectable, ns, not significant.

To study the contribution of LRAT to intestinal retinoid metabolism, we performed studies in LRAT-deficient mice and WT mice (28). We subjected mice to dietary vitamin A deprivation for 1 week to deplete the intestine from retinoids. After the washout phase, mice were randomized into two groups, which received either diet supplemented with retinyl acetate [vitamin A-sufficient (VAS) diet group] or BC (BC group). After the 3-day dietary intervention, we determined retinoid concentrations in jejunal lipid extracts by quantitative HPLC analysis (Fig. 1A, B). ROL was detectable in all mice, indicating that enterocytes absorbed and metabolically processed dietary retinoid precursors. ROL concentration was highest in WT mice (0.4 nmol g^−1^) and lowest in BC-supplemented *Lrat*^*−/−*^ mice (0.1 nmol g^−1^) (Fig. 1C). RE concentration (0.9 nmol g^−1^) was 5-fold higher in WT mice than in *Lrat*^*−/−*^ mice of the VAS group, confirming the importance of LRAT for intestinal RE synthesis (Fig. 1D) (27). The remaining RE in *Lrat*^*−/−*^ mice of the VAS group was likely synthesized by coenzyme A-dependent ROL acyltransferases (30). RE was absent in *Lrat*^*−/−*^ mice of the BC group (Fig. 1D). Instead, the intestinal lipid extracts from these mice contained significant amounts of BC (Fig. 1E).

### The transcription factor ISX is hypersensitive to dietary vitamin A precursors in LRAT-deficient mice

**Figure 2:**
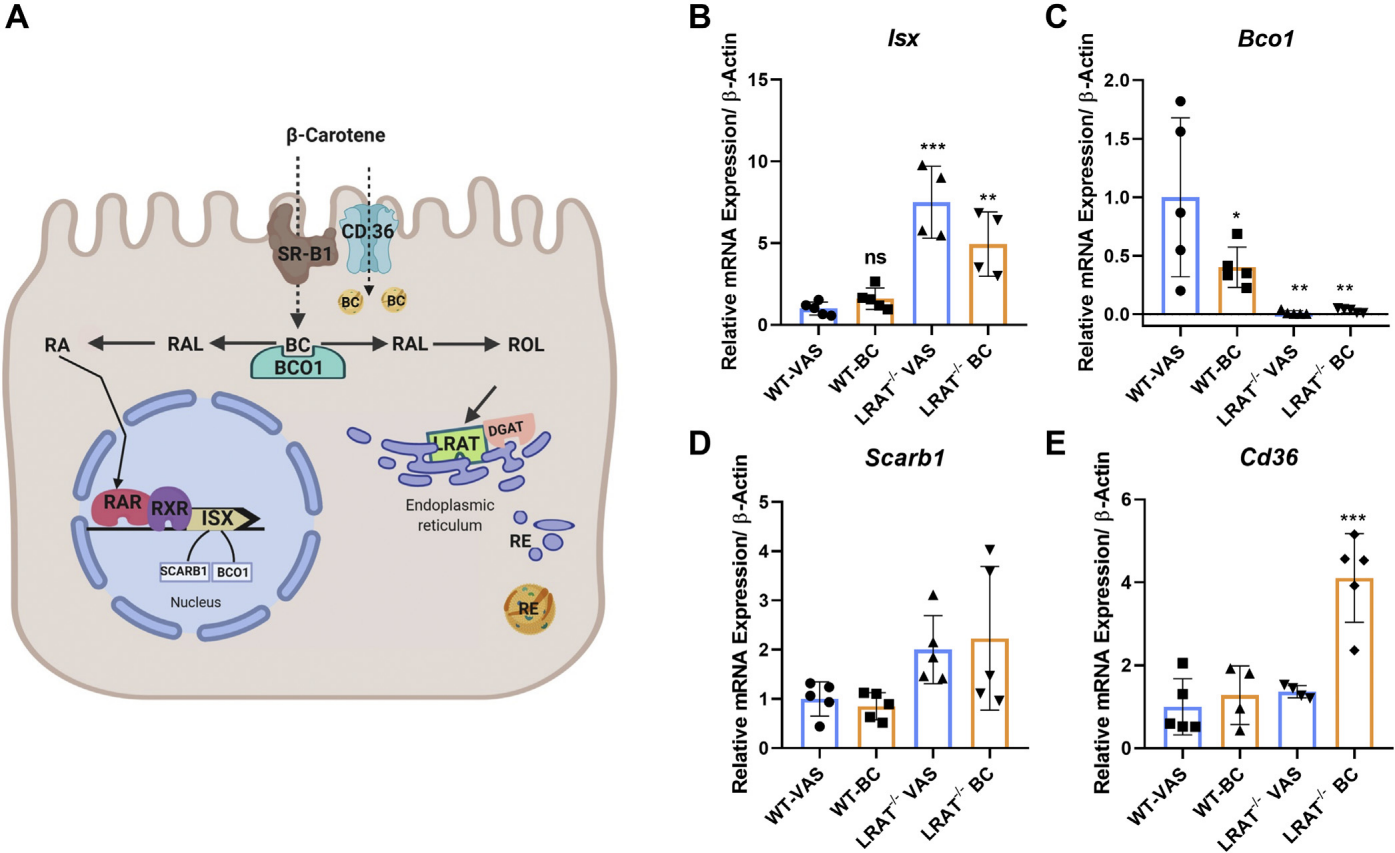
mRNA levels of marker genes for retinoid biosynthesis in the jejunum of *Lrat*^*−/−*^ and WT mice on different diets. WT and *Lrat*^*−/−*^ mice (*n* = 4–5 per genotype) were fed with diets supplemented with vitamin A [vitamin A sufficiency (VAS)] and β-carotene (BC). Jejunums were dissected, and total RNA was isolated and subjected to quantitative RT-PCR analysis for *Isx*, *Bco1*, *Scarb1*, and *Cd36* mRNA levels. A: Scheme of components involved in retinoid biosynthesis and its regulation in intestinal enterocytes. B: *Isx* mRNA levels. C: *Bco1* mRNA levels. D: *Scarb1* mRNA levels. E: *Cd36* mRNA levels. β-actin was used as an internal control. Data were normalized to the mRNA levels in WT mice on VAS diet and displayed as means ± SD. ∗*P* < 0.05, ∗∗*P* < 0.005, and ∗∗∗*P* < 0.0001. The statistical analysis was performed using one-way ANOVA by comparing WT-VAS as control. ISX, intestine-specific homeodomain transcription factor; LRAT, lecithin:retinol acyltransferase; ns, not significant; RAL, retinal; RAR, retinoic acid receptor; RE, retinyl ester; ROL, retinol; RXR, retinoid X receptor.

We used quantitative RT-PCR to determine the relative mRNA levels of genes encoding proteins involved in retinoid biosynthesis and its regulation in enterocytes (Fig. 2A). Analyses of intestinal RNA preparations revealed that mRNA levels of the transcription factor ISX were 4- to 8-fold higher in *Lrat*^*−/−*^ mice when compared with that in WT mice (Fig. 2B). Conversely, mRNA levels of the ISX-target gene *Bco1* were 20-fold lower in *Lrat*^*−/−*^ mice when compared with WT mice of the VAS group (Fig. 2C). *Scarb1* mRNA expression levels did not follow this trend and were comparable between the two genotypes (Fig. 2D). The mRNA level of *Cd36* gene was 4-fold higher in *Lrat*^*−/−*^ mice of the BC group than in WT mice of the VAS group (Fig. 2E). This scavenger receptor may provide an alternative route for BC uptake from the diet (31).

### LRAT-deficient mice display impaired BC metabolism

We observed that the transcription factor ISX became hypersensitive to dietary retinoid precursors and suppressed *Bco1* gene expression in LRAT-deficient enterocytes. To explore the long-term consequences of this phenotype, we subjected *Lrat*^*−/−*^ mice to a 5-week-long dietary intervention study. After a 1-week washout phase to deprive them from dietary vitamin A, we randomized mice into three experimental groups. One group of mice received VAD diet and served as baseline control (VAD group), the second group received diet supplemented with preformed vitamin A (VAS group), and the third group received diet supplemented with provitamin A (BC group).

**Figure 3:**
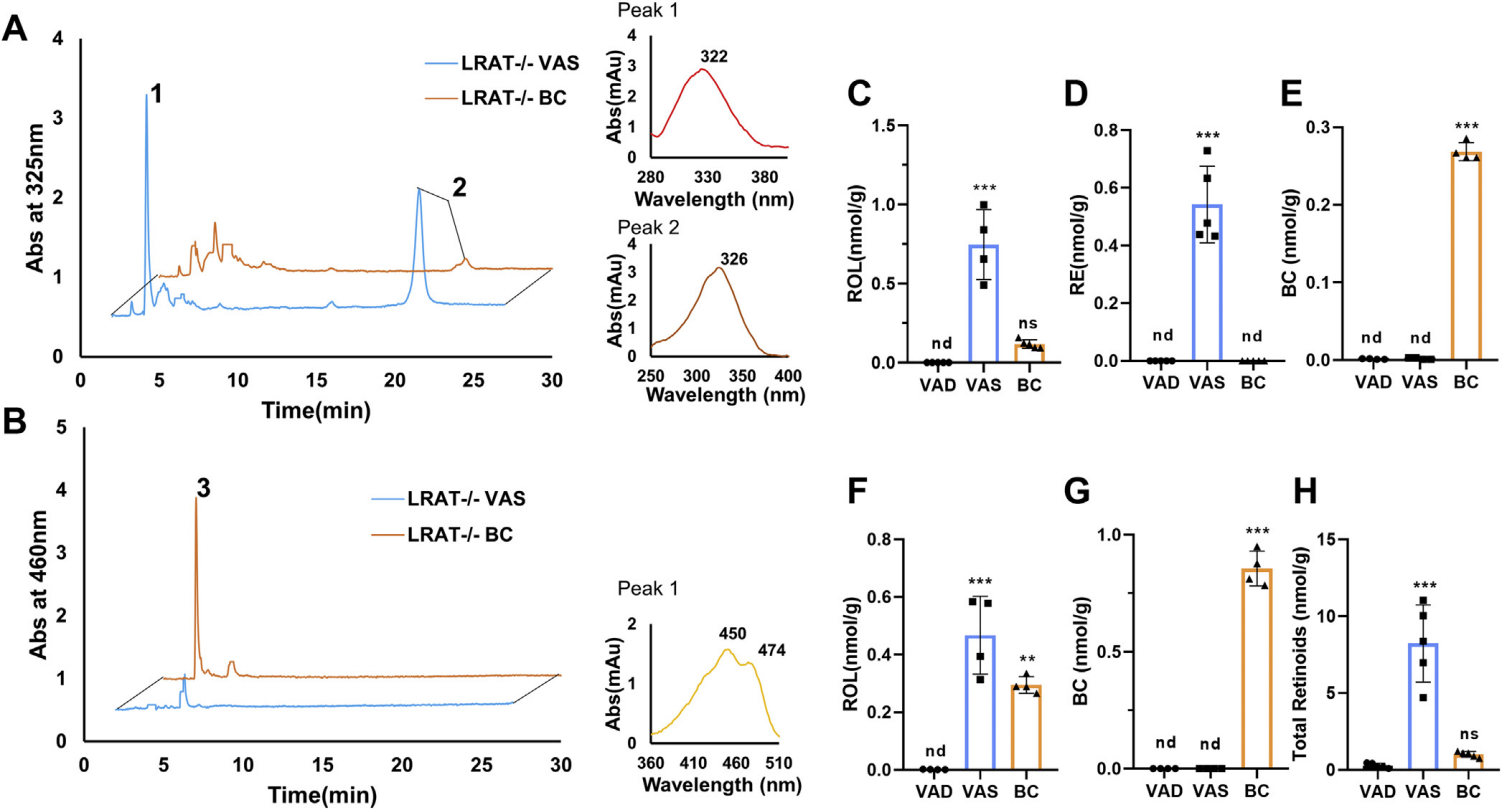
Long-term feeding of β-carotene (BC) disrupts vitamin A homeostasis in *Lrat*^*−/−*^ mice. *Lrat*^*−/−*^ mice (*n* = 4–5 animals per dietary intervention group) were subjected to feeding with either vitamin A-deficient (VAD) diet, vitamin A-sufficient (VAS) diet, and BC diet for 4 weeks. A and B: HPLC chromatograms at 325 nm (A) and 460 nm (B) of jejunal lipid extracts. Peak 1 represents retinyl esters (REs), peak 2 represents all-*trans*-retinol (ROL), and peak 3 represents BC. Spectral characteristics of peaks are shown on the right. C–E: Quantification of ROL, RE, and BC concentrations. F and G: Hepatic ROL and BC concentrations. H: Total retinoid concentration in gonadal adipose depots. The data represent means ± SD. ∗*P* < 0.05, ∗∗*P* < 0.005, and ∗∗∗*P* < 0.0001. The statistical analysis was performed using one-way ANOVA by comparing VAD-fed mice as control. nd, not detectable; ns, not significant.

After the dietary intervention, we determined retinoid and BC levels in jejunum and liver of mice in different groups by HPLC analyses (Fig. 3A, B). In addition, we determined retinoid concentration in the gonadal white adipose tissues where *Lrat*^*−/−*^ mice store significant amounts of retinoids (27). The quantified concentrations of ROL, RE, and BC in the different tissues are displayed in Fig. 3. As expected, BC, ROL, and RE were absent in the jejunum of the VAD baseline group, which received no vitamin with the diet (Fig. 3C–E). In the VAS group, both ROL and RE (0.8 and 0.75 nmol g^−1^) became detectable in the intestine, as indicated by their characteristic retention times and spectral characteristics on the HPLC system (Fig. 3C, D). ROL was 5-fold lower in mice of the BC group when compared with VAS group (Fig. 3C). RE, the transport form of vitamin A, was below the detection levels of the HPLC system in the intestine of the BC group (Fig. 3D). In contrast, BC (0.26 nmol g^−1^) became detectable in lipid extracts of the intestine, indicating that the provitamin was absorbed but not converted to ROL and RE (Fig. 3E).

Mice of the VAS group also displayed the highest hepatic concentration of ROL (0.6 nmol g^−1^) (Fig. 3F). Mice of the BC group displayed about half of this concentration, whereas ROL was absent in hepatic lipid extracts of the VAD group (Fig. 3F). Mice of the BC group accumulated significant concentrations of BC (0.8 nmol g^−1^) in the liver, whereas the provitamin was absent in the other groups (Fig. 3G). In gonadal adipose depots, mice of the VAS group displayed significant vitamin A stores (8 nmol g^−1^) (Fig. 3H). In the BC group, the stores were 8-fold lower than that in the VAS group and barely surmounted the total retinoid concentration of the VAD baseline group (Fig. 3H). Interestingly, BC concentrations were below the detection level of the HPLC system in gonadal adipose tissues of *Lrat*^*−/−*^ mice. These biochemical analyses showed that LRAT mice fed a BC-rich diet absorbed significant amounts of the provitamin A intact and displayed reduced retinoid concentrations in liver and fat when compared with siblings supplemented with preformed vitamin A (VAS group).

### Dietary BC cannot maintain retinoid-dependent processes in gastrointestinal immunity in *Lrat*^*−/−*^ mice

**Figure 4:**
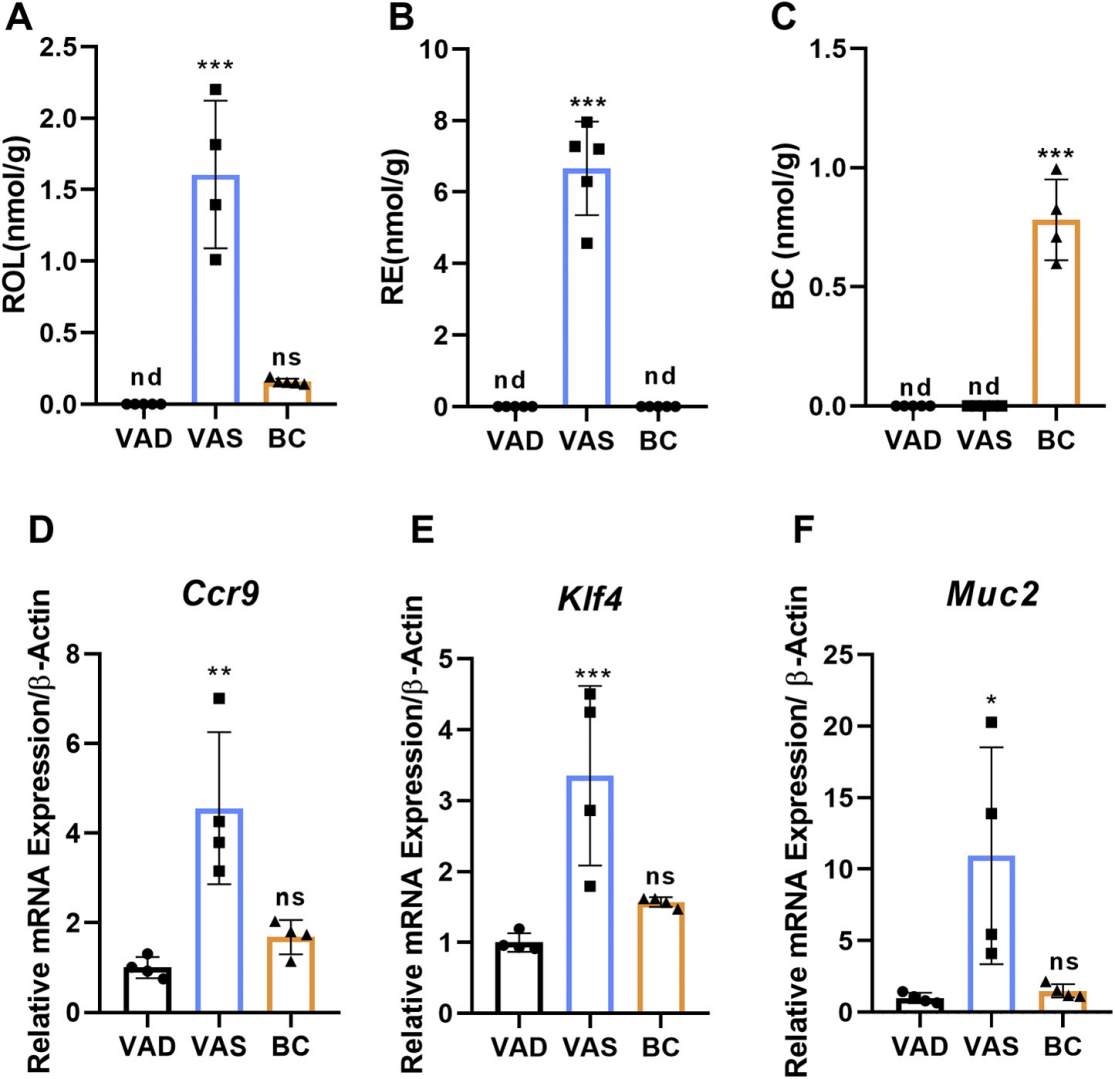
Provitamin A supplementation alters mesenteric lymph node retinoid homeostasis and intestinal immune cell markers in *Lrat*^*−/−*^ mice. *Lrat*^*−/−*^ mice (*n* = 4–5 animals per dietary intervention group) were subjected to feeding with vitamin A-deficient (VAD) diet, vitamin A-sufficient (VAS) diet, and β-carotene (BC) diet for 4 weeks. Mesenteric lymph nodes were dissected and subjected to analysis of nonpolar retinoids and BC. A: ROL concentration. B: RE concentrations. C: BC concentrations. D–F: Quantitative RT-PCR analysis with total RNA preparations of the jejunum for *Ccr9*, *Klf4*, and *Muc2* mRNA levels. β-actin was used as internal control. Values were normalized to the mRNA levels of mice of the VAD group and are displayed as means ± SD. ∗*P* < 0.05, ∗∗*P* < 0.005, and ∗∗∗*P* < 0.0001. The statistical analysis was performed using one-way ANOVA by comparing VAD-fed mice as control. ns, not significant; RE, retinyl ester; ROL, retinol.

Vitamin A is critical for the maintenance of gastrointestinal immunity and barrier function (20, 32). To determine how different diets affect these processes in *Lrat*^*−/−*^ *mice*, we determined retinoid levels in isolated mesenteric lymph nodes by HPLC analysis. We measured significant amounts of ROL and RE (1.5 and 6 nmol g^−1^) in mice of the VAS group (Fig. 4A, B), indicating that lymphoid cells of LRAT-deficient mice can acquire vitamin A when supplemented with preformed vitamin A. In contrast, highly diminished concentrations of ROL and RE were detected in mesenteric lymph node of mice in the BC group. Instead, mice of the BC group accumulated the provitamin A (0.8 nmol g^−1^) in their mesenteric lymph nodes (Fig. 4C). As expected, retinoids were absent in the lymph nodes of mice of the VAD group (Fig. 4A, B). As additional control, we determined the levels of retinoids in mesenteric lymph nodes of WT mice raised for 6 weeks on VAS and BC diets. HPLC analysis detected comparable levels of ROL and RE under both dietary conditions, indicating that preformed vitamin A and BC can maintain vitamin A homeostasis in this organ in WT mice (supplemental Fig. S1). In addition, ROL and RE concentrations were within the same range as in *Lrat*^*−/−*^ mice of the VAS group.

To examine the effects of different diets on immune cells in *Lrat*^*−/−*^ mice, we determined mRNA expression levels of the CCR9, which is expressed in response to RA signaling and promotes gut homing of B and T cells (33). We observed 4-fold higher levels of *Ccr9* mRNA in the jejunum of the VAS group when compared with BC and VAD groups (Fig. 4D). It has long been known that vitamin A affects barrier function of the intestine, including goblet cells (34). Therefore, we determined mRNA levels of KLF4, a marker gene of these cells (35). In addition, we measured mRNA levels of the *mucin-2* gene. The encoded protein is secreted from goblet cells in order to form a protective luminal mucus layer (36), and its expression is regulated by retinoid signaling (37). The analyses revealed that mRNA levels of *Klf4* and *mucin-2* were respectively 3-fold and 10-fold lower in mice of the BC and VAD groups when compared with mice of the VAS group (Fig. 4E, F). Thus, BC diet similar to VAD diet was not able to maintain retinoid-dependent processes at the intestinal barrier of LRAT-deficient mice.

### Expression levels of retinoid-responsive genes in the gut and liver of LRAT-deficient mice

**Figure 5:**
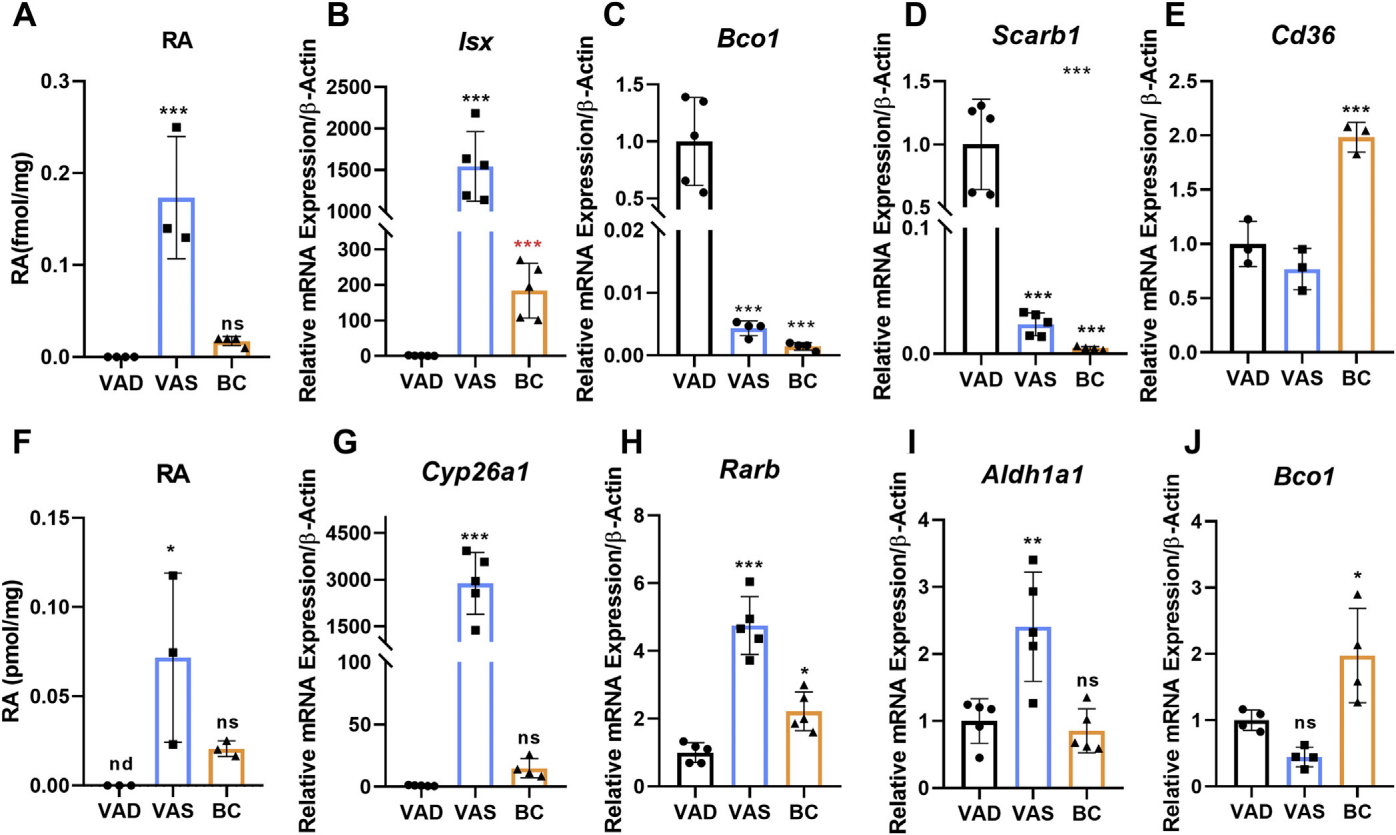
Retinoic acid (RA) concentrations and mRNA levels of retinoid responsive genes in the LRAT-deficient intestine and liver. *Lrat*^*−/−*^ mice (*n* = 4–5 animals per dietary intervention group) were subjected to feeding with either vitamin A-deficient (VAD) diet, vitamin A-sufficient (VAS) diet, and β-carotene (BC) diet for 4 weeks. A: Intestinal RA concentrations in different dietary groups. B–E: Quantitative RT-PCR analysis for *Isx*, *Bco1*, *Scarb1*, and *Cd36* mRNA levels was performed with total RNA preparation of the jejunum of different groups. B: *Isx* mRNA levels. (BC-supplemented mice were not significant for one-way ANOVA test). The red ∗∗∗ represent the statistical analysis for Student's *t*-test analysis. C: *Bco1* mRNA levels. D: *Scarb1* mRNA levels. E: *Cd36* mRNA levels. β-actin was used as internal control. Data were normalized to the mRNA levels of *Lrat*^*−/−*^ mice on VAD diet. F: Hepatic RA concentrations in different dietary groups. G–J: Quantitative RT-PCR analysis for *Cyp26a1*, *Rarb*, *Aldh1a1*, and *Bco1* mRNA levels was performed with total RNA preparation of the livers of different groups. G: *Cyp26a1* mRNA levels. H: *Rarb* mRNA levels. I: *Aldh1a1* mRNA levels. J: *Bco1* mRNA levels. β-actin was used as internal control. Data were normalized to the mRNA levels of *Lrat*^*−/−*^ mice of the VAD group and are displayed as means ± SD. ∗*P* < 0.05, ∗∗*P* < 0.005, and ∗∗∗*P* < 0.0001. The statistical analysis was performed using one-way ANOVA by comparing VAD-fed mice as control. nd, not detectable; ns, not significant.

To investigate the molecular basis of impaired BC metabolism in *Lrat*^*−/−*^ mice, we performed LC/MS analysis for RA and determined RNA levels for maker genes of retinoid signaling. LC/MS analysis detected RA (0.18 pmol g^−1^) in intestinal lipid extracts of mice of the VAS group (Fig. 5A and supplemental Fig. S2). Though mice of the BC group displayed highly reduced ROL and RE concentrations (Fig. 3C, D), RA (0.05 pmol g^−1^) became detectable in their intestinal lipid extracts (Fig. 5A). As expected, RA was absent in the VAD group, which received no vitamin A precursor with the diet (Fig. 5A). The measurements of mRNA levels of *Isx* reflected the RA concentration in different dietary intervention groups (Fig. 5B). *Isx* mRNA levels were 1,500-fold higher in the VAS group and 180-fold higher in the BC group when compared with the VAD baseline group (Fig. 5B). The mRNA levels of the ISX target genes *Bco1* and *Scarb1* displayed a reverse pattern of expression, being several 100-fold lower in mice of the VAS and BC groups when compared with the VAD group (Fig. 5C, D). Mice of the BC group also showed 2-fold higher levels of *Cd36* mRNA when compared with the VAS and VAD intervention groups (Fig. 5E).

In the liver, RA levels were high in the VAS group (0.07 pmol g^−1^), low in the BC group (0.02 pmol g^−1^), and absent in the VAD group (Fig. 5F). The expression patterns of target genes of canonical retinoid signaling paralleled hepatic RA concentrations. The mRNA levels of *Cyp26a1*, encoding an RA-inducible retinoid hydroxylase (38), were 3,000-fold and 15-fold higher in the VAS and BC groups, respectively, when compared with the VAD baseline group (Fig. 5G). Other marker genes of hepatic retinoid metabolism such as *Rarb* and the RA synthesizing enzyme *Aldha1* displayed similar patterns of expression as *Cyp26a1* (Fig. 5H, I). However, the differences between dietary groups were far less pronounced when compared with *Cyp26a1* mRNA (Fig. 5G). We also determined the expression levels of the BC converting enzyme BCO1. Though the provitamin accumulated in the liver, mice of the BC group expressed *Bco1* mRNA in this organ (Fig. 5J).

### Pharmacological inhibition of retinoid signaling restores *Bco1* expression in LRAT-deficient enterocytes

**Figure 6:**
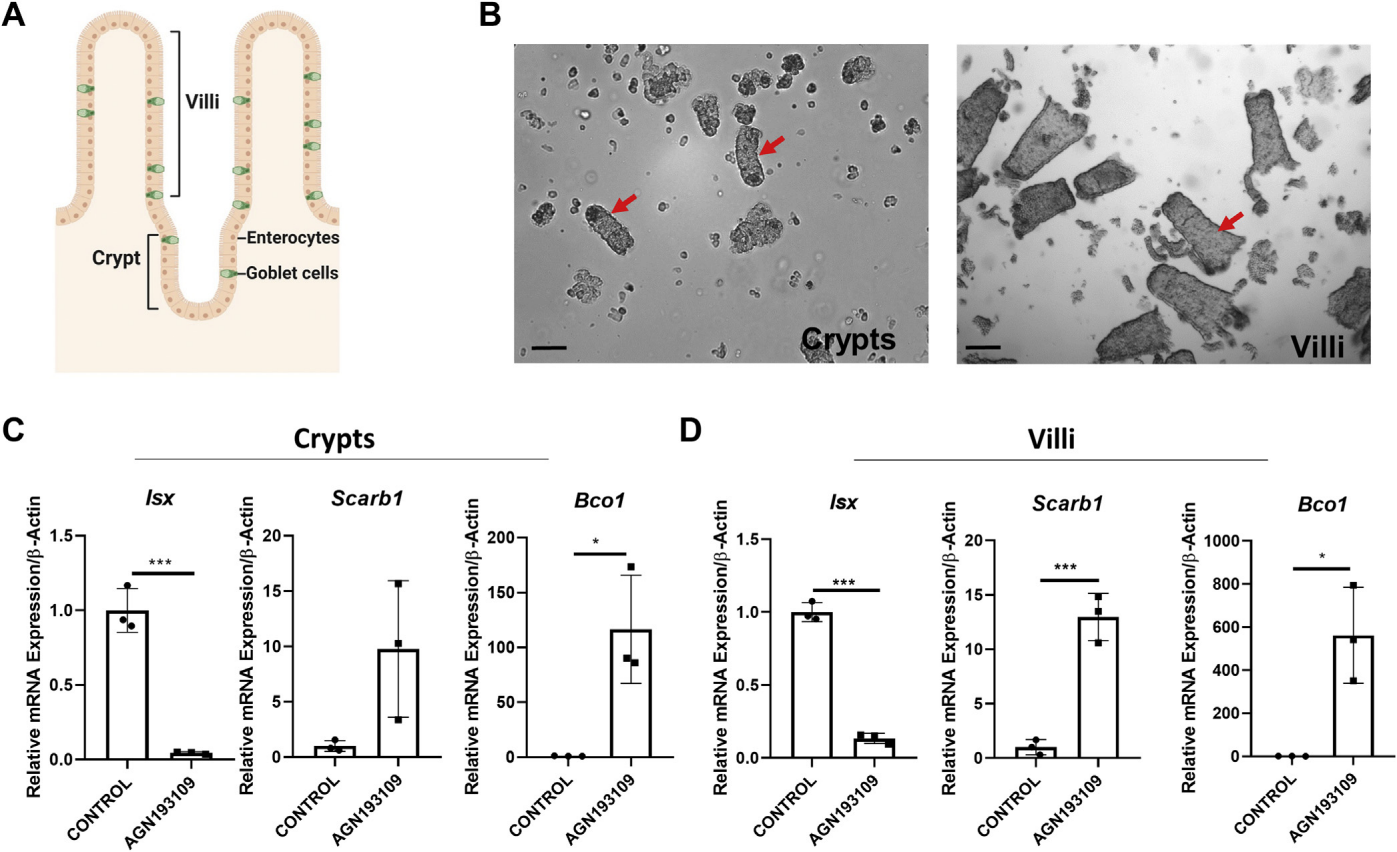
Pharmacological intervention with a pan-retinoic acid receptor (RAR) antagonist decreases *Isx* mRNA expression and restores *Bco1* mRNA expression. *Lrat*^*−/−*^ mice (*n* = 3 per condition) were subjected to β-carotene diet and treated with pan-RAR antagonist (AGN193109) or vehicle alone as outlined in Materials and methods section. A: Scheme of intestinal villi and crypts. B: Representative images of isolated villi and crypts of the intestine (red arrows). Images were taken at 8× magnification. The scale bar represents 100 μm. C and D: Quantitative RT-PCR (qRT-PCR) analysis of *Isx*, *Scarb1*, and *Bco1* mRNA levels in vehicle (control) and pan-RAR antagonist (AGN193109) treated mice. C: qRT-PCR analysis of *Isx*, *Scarb1*, and *Bco1* mRNA expression in crypts. D: qRT-PCR analysis of *Isx*, *Scarb1*, and *Bco1* mRNA expression in villi. Values were normalized to the mRNA levels of mice of control group and are displayed as means ± SD. ∗*P* < 0.05, ∗∗*P* < 0.005, and ∗∗∗*P* < 0.0001 in unpaired two-tailed *t*-test.

We previously showed in human Caco-2 cells, a cell line model for enterocytes, that RARs regulate *Isx* gene expression (16). Thus, we expected that pharmacological inhibition of retinoid signaling by the antagonist would decrease the expression of the transcription factor and restore *Bco1* mRNA expression in the LRAT-deficient intestine. For this purpose, we subjected *Lrat*^*−/−*^ mice to BC supplementation for 1 week. Then, we administered a pan-RAR antagonist AGN193109 twice by oral gavage in intervals of 24 h. We analyzed mice 6 h after the second dose of the drug. For this purpose, we dissected the intestine and isolated villi and crypts by an established protocol. We then isolated RNA from the cell preparations (Fig. 6A, B) and determined the expression levels of the *Isx*, *Bco1*, and *Scarb1* genes. *Isx* mRNA levels were 25-fold and 10-fold higher in crypts and villi of the control group when compared with antagonist-treated mice (Fig. 6C, D). Conversely, antagonist-treated mice revealed 100-fold and 500-fold higher levels of *Bco1* mRNA in crypts and villi, when compared with vehicle-treated mice. Similarly, *Scarb1* mRNA levels were 9-fold and 12-fold higher in crypts and villi of antagonist-treated mice when compared with vehicle-treated mice (Fig. 6C, D). Thus, a pan-RAR antagonist can block ISX signaling and restore *Bco1* mRNA expression in the intestine of BC-supplemented *Lrat*^*−/−*^ mice.

### Genetic rescue of retinoid biosynthesis in LRAT-deficient mice

**Figure 7:**
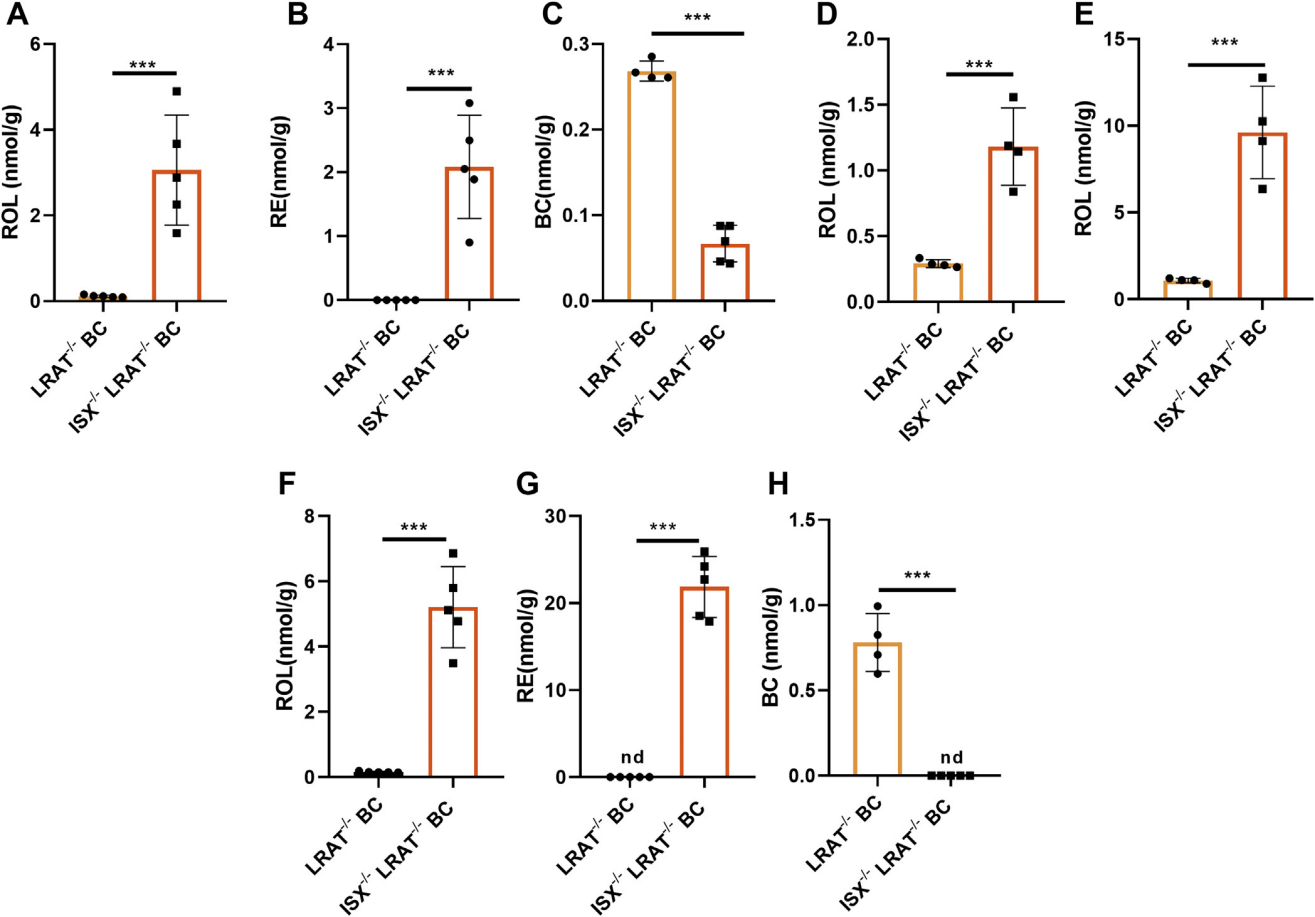
Genetic depletion of *Isx* gene restores retinoid biosynthesis in *Lrat*^*−/−*^ mice. *Lrat*^*−/−*^ and *Lrat*^*−/−*^*/Isx*^*−/−*^ (double knockout) mice (*n* = 4–5 per genotype) were fed with β-carotene (BC) diet for 4 weeks. A–C: Quantification of the concentration of all-*trans*-retinol (ROL), retinyl esters (REs), and β-carotene (BC) in jejunum of the mice. D and E: ROL concentration of the liver and the gonadal adipose depots. F–H: Quantification of the concentration of ROL, RE, and BC of mesenteric lymph nodes. The data represent means and ± SD. ∗∗∗*P* < 0.0001 using unpaired two-tailed *t*-test comparing each genotype. nd, not detectable.

We next wanted to demonstrate that the suppression of *Bco1* gene activity in LRAT-deficient enterocytes is ISX dependent. Therefore, we performed a genetic rescue experiment and generated an *Isx*^*−/−*^, *Lrat*^*−/−*^, DKO mouse mutant by conventional crossings. We expected that the depletion of the *Isx* gene would rescue *Bco1* gene expression and retinoid biosynthesis in DKO mice. Thus, we subjected DKO and *Lrat*^*−/−*^ mice to dietary intervention with BC as the sole source for retinoids. After 4 weeks, we determined retinoid concentration in the jejunum, liver, and adipose tissue of mice to assess the vitamin A status of the single- and double-mutant mice. Quantitative HPLC analyses revealed that DKO mice displayed high levels of ROL and RE (3 and 2 nmol g^−1^, respectively) in the intestine, whereas retinoids were barely detectable in *Lrat*^*−/−*^ mice (Fig. 7A, B). Conversely, *Lrat*^*−/−*^ mice displayed higher levels of BC when compared with DKO mice (0.27 vs. 0.03 nmol g^−1^). ROL concentrations were respectively 3-fold and 8-fold higher in DKO mice than in *Lrat*^*−/−*^ mice in the liver and white adipocytes, respectively (Fig. 7D, E). In addition, the DKO mice displayed high levels of ROL and RE in mesenteric lymph nodes (7 and 20 nmol g^−1^, respectively), demonstrating that the genetic deletion of *Isx* gene restored vitamin A delivery to immune cells in LRAT deficiency (Fig. 7F, G). Notably, DKO mice did not accumulate BC in lymph nodes, whereas *Lrat*^*−/−*^ mice again showed this accumulation (Fig. 7G). The outcome of the biochemical analysis was mirrored in *Bco1* and *Scarb1* mRNA levels, which were more than 100-fold higher in the intestine of DKO mice when compared with *Lrat*^*−/−*^ mice (supplemental Fig. S3A, B). In the liver, *Cyp26a1* and *Rarb* mRNA levels were 900-fold and 4-fold increased in DKO mice when compared with *Lrat*^*−/−*^ mice (supplemental Fig. S3C, D). Thus, our analyses showed that depletion of the *Isx* gene restored *Bco1* gene expression and retinoid biosynthesis in LRAT-deficient enterocytes and improved the vitamin A status of the animals.

## Discussion

The biosynthesis of retinoids from dietary BC is a classic example of a diet-responsive negative-feedback loop. By default, SR-B1 and BCO1 proteins acquire and convert carotenoids to retinoids whenever they become available in the diet. However, enterocytes very sensibly cease the expression of the corresponding genes in response to retinoid signaling. This mechanism ensures optimal retinoid levels in the body and avoids excessive production of these compounds on diets rich in BC (19). In the current work, we analyzed how the enterocytes regulate retinoid concentrations in this negative feedback loop and adapt them to the bodily requirement. We demonstrate in mouse models that the catalytic activity of LRAT in enterocytes is central to the control of this lipid-based signaling cascade.

LRAT is a member of the N1pC/P60 multigene family of integral membrane proteins. These proteins perform catalysis at the lipid membrane-water interface and use phospholipids as substrates (39). During LRAT catalysis, a thioacyl enzyme intermediate forms, and the acyl group transfers from its thioester bond to ROL. For this purpose, LRAT displays a specific retinoid-binding domain (26), which first evolved in jawless vertebrates (40). LRAT has been shown to be critically involved in cellular uptake of ROL from serum ROL-binding protein into peripheral tissues (41), storage of retinoids in liver and lung (27), as well as visual chromophore synthesis and recycling (28). We herein describe a novel function of LRAT in the control of intestinal retinoid biosynthesis from dietary BC. Using knockout mouse models, we observed that LRAT-deficient animals were not able to properly utilize dietary BC for retinoid biosynthesis. *Lrat*^*−/−*^ mice on BC diet displayed reduced ROL and RE levels in the intestine, liver, and fat when compared with siblings fed with preformed vitamin A. We provide evidence that this phenotype is caused by a dysregulation of the signaling through the transcription factor ISX in the LRAT-deficient intestine. We demonstrate that *Lrat*^*−/−*^ mice displayed higher expression levels of the transcription factor than WT mice. Our analysis indicates that this phenotype is caused by increased RA production from dietary vitamin A precursors in the LRAT-deficient intestine. Enhanced retinoid signaling and increased activity of the transcription factor ISX resulted in a suppression of BCO1 activity that prevented BC conversion to retinoids. This assumption was corroborated by the pharmacological studies where treatment with a RAR antagonist restored *Bco1* expression in LRAT-deficient enterocytes. Similarly, genetic depletion of the *Isx* gene restored retinoid biosynthesis in an ISX- and LRAT-deficient double-mutant mouse line. In fact, the DKO mice efficiently converted BC to retinoids and distributed them to extrahepatic tissues. These findings demonstrate that dysregulation of *Isx* expression and subsequent suppression of *Bco1* gene expression constituted the molecular basis for reducing the retinoid biosynthesis from BC in the LRAT-deficient intestine. Our findings also demonstrate that *Bco1* gene expression is directly controlled through ISX and not through an interaction between RARs and the *Bco1* gene promoter as previously proposed by others (14). Thus, we conclude that the catalytic activity of LRAT is required to balance intestinal retinoid concentrations and to ensure that the majority of absorbed BC is converted to RE for bodily distribution.

We previously reported that gastrointestinal immunity is severely impaired in ISX-deficient mice when subjected to BC feeding (42). We now observed that the different diets had a profound effect on gastrointestinal immunity and barrier function in LRAT-deficient mice. *Lrat*^*−/−*^ mice on BC diet displayed highly reduced retinoid stores in mesenteric lymph nodes. In contrast, WT mice were able to maintain their retinoid levels when fed with BC. The biochemical phenotype of LRAT-deficient mice of the BC group was associated with decreased expression of *Ccr9*, a marker gene for gastrointestinal lymphocytes. Furthermore, reduced *Klf4* and *mucin-2* expression indicated that goblet cell function was altered in *Lrat*^*−/−*^ mice on BC diet. A similar reduction of *Klf4* and *mucin-2* gene expression was observed in *Lrat*^*−/−*^ mice subjected to dietary vitamin A depletion, indicating that the effects are caused by reduced retinoid concentrations and not exerted by BC and/or long-chain apocarotenoids.

The previous and present findings indicate that RA levels at the intestinal barrier must be tightly controlled to maintain a physiological state. In the intestine, RA has a broad spectrum of effector functions and, under specific conditions, RA can promote an inflammatory environment (43). Common genetic variability in the general population affects vitamin A status and bioavailability (44). Therefore, more research is needed to elucidate the details of intestinal retinoid metabolism and the putative consequence of genetic variability in the involved genes. Additional study is required to clarify how involved components affect immune cell differentiation and function. In addition, the putative role of these components on cell differentiation needs to be addressed because *Isx* and *Bco1* are expressed in crypts (17), the stem cell niche of the gut epithelium.

We also observed that LRAT-deficient mice accumulated intact BC in the liver and lymph nodes. Children receiving vitamin A supplements and consuming BC-rich fruits display a similar carotenemia (45). In a gerbil model, oversupplementation with these micronutrients resulted in reduced *Bco1* expression in the intestine and BC accumulation as well (46), indicating that suppression of intestinal BCO1 activity results in BC accumulation in the body. Interestingly, we observed that *Cd36* gene expression was elevated in the intestine of BC-supplemented *Lrat*^*−/−*^ mice. Thus, we propose that CD36 facilitates the uptake of BC in enterocytes under this condition.

The Ross laboratory showed that LRAT is expressed at high levels in the rodent intestine (47). This mode of expression parallels *Bco1* and *Scarb1* expression and ensures an efficient retinoid biosynthesis from BC. In contrast to the *Scarb1* and *Bco1*, ISX does not suppress *Lrat* gene expression in enterocytes (15). The absence of this regulation might be an evolutionary adaptation to diets with high vitamin A content. LRAT transforms ROL into its inert ester form and prevents toxic and teratogenic effects of the nutrient, as we previously showed in a zebrafish model (24). In humans, hypervitaminosis A occurs rarely from the consumption of regular diets. However, there are published reports from early last century indicating that arctic explorers experienced acute vitamin A toxicity after they consumed polar bear or seal liver (48). These animals are on the top of the food chain and contain 10–20 times higher levels of the vitamin in their livers than other artic animals or their genetically related predators in moderate climates (49). The development of such an efficient vitamin A-storing mechanism in hepatic stellate cells contributed to the survival of these top predators in the extreme environment of the arctic.

In conclusion, we here reported that the catalytic activity of the LRAT enzyme plays a critical role in the control of retinoid biosynthesis in the mouse intestine. In this process, LRAT dynamically adjusts the concentration of retinoids by sequestering RE for body distribution and storage. This homeostatic mechanism ensures adequate regulation of BCO1 activity and portioning of retinoids in the body to maintain physiological processes such as vision, immunity, and cellular differentiation.

## Data availability

The authors confirm that the data supporting the findings of this study are contained within the article. The raw data are available upon request from the corresponding author (Dr J. v. L., Department of Pharmacology, Case Western Reserve University; E-mail: johannes.vonlintig@case.edu).

## Supplemental data

This article contains supplemental data.

## Conflict of interest

The authors declare that they have no conflicts of interest with the contents of this article.

## Supplemental data

Supp lemental Figures S1 to S3
