# Supplementary material for: LRAT coordinates the negative-feedback regulation of intestinal retinoid biosynthesis from β-carotene
Source: J Lipid Res. 2021 Feb 23;62:100055. doi: 10.1016/j.jlr.2021.100055 (PMC8010212; doi:10.1016/j.jlr.2021.100055)
Supplement: Supp — lemental Figures S1 to S3 [file mmc1.doc]

**Supporting Information**

**
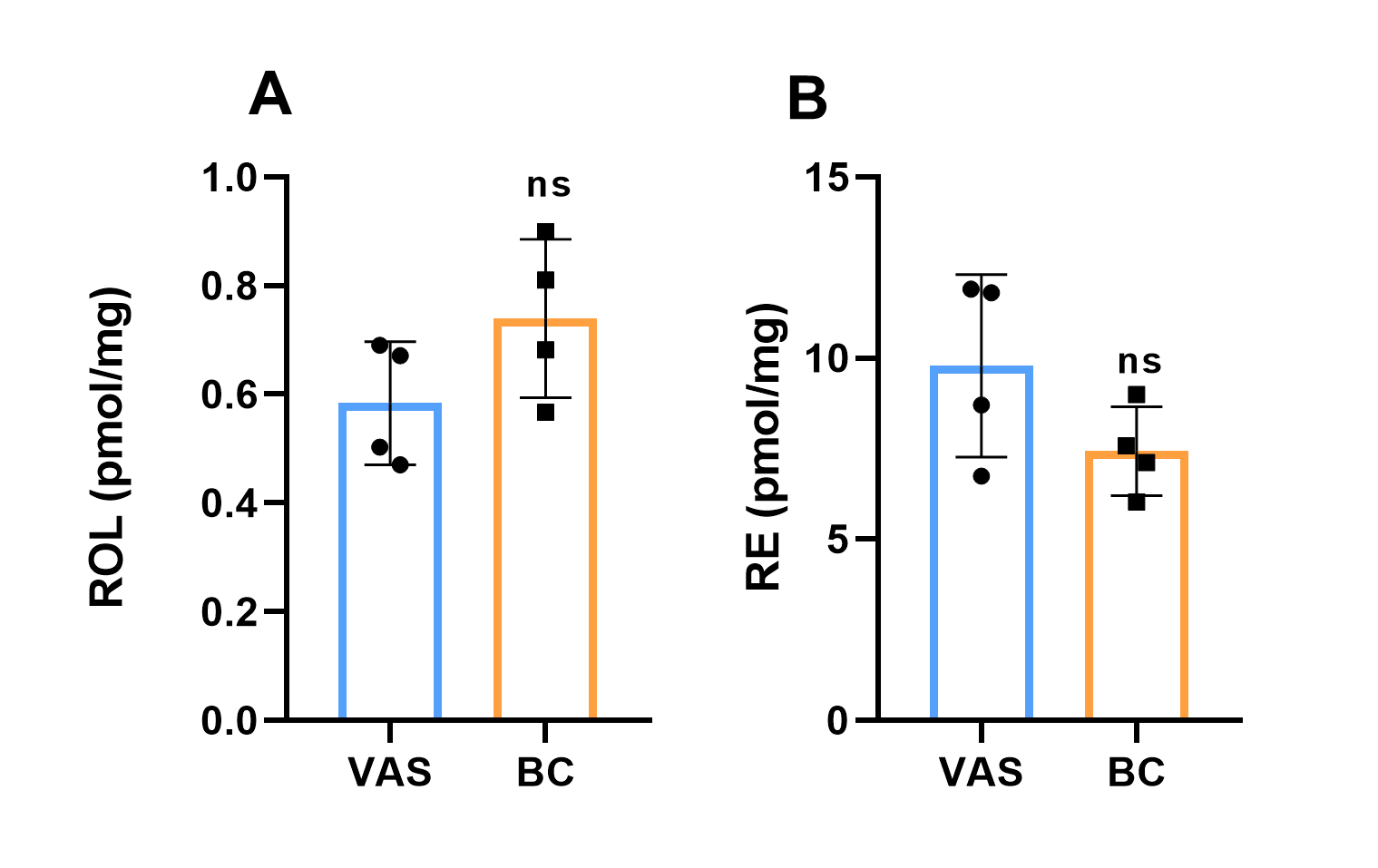
**

Figure S1: **Mesenteric lymph node retinoid analysis in wild type mice**. WT mice (n= 4 animals per dietary intervention group) subjected to feeding with vitamin A sufficient diet (VAS), and β-carotene (BC) diet. (A, B) ROL and RE concentration of mesenteric lymph node respectively. The data represent means and ±SD. The statistical analysis was carried out using unpaired two tail *t* test. ns, not significant

**
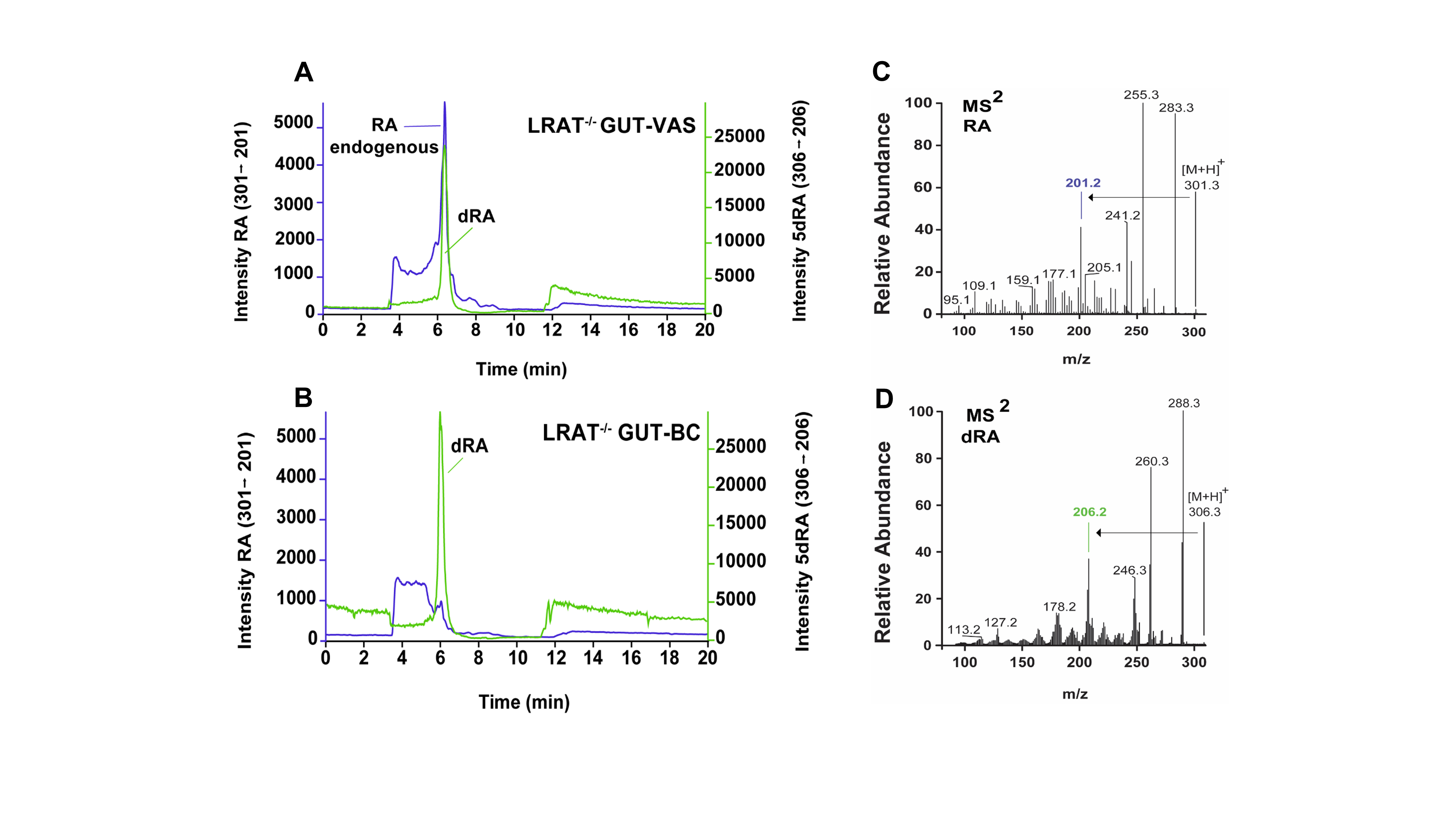
**

**Supplementary Figure 2.** A representative LC/MS/MS profile for retinoic acid from an intestinal lipid extract obtained from VAS and BC diet fed *Lrat^-/-^* mice. (A,B) *Lrat^-/-^  mouse intestine* extracts showing the MRM peaks of all-*trans*-retinoic acid (blue trace RA) and deuterated all-trans-retinoic acid internal standard (green trace). (C) MS^2^ fragmentation of endogenous all-trans-retinoic acid. (D) MS^2^ fragmentation of the deuterated all-trans-retinoic acid standard.

**
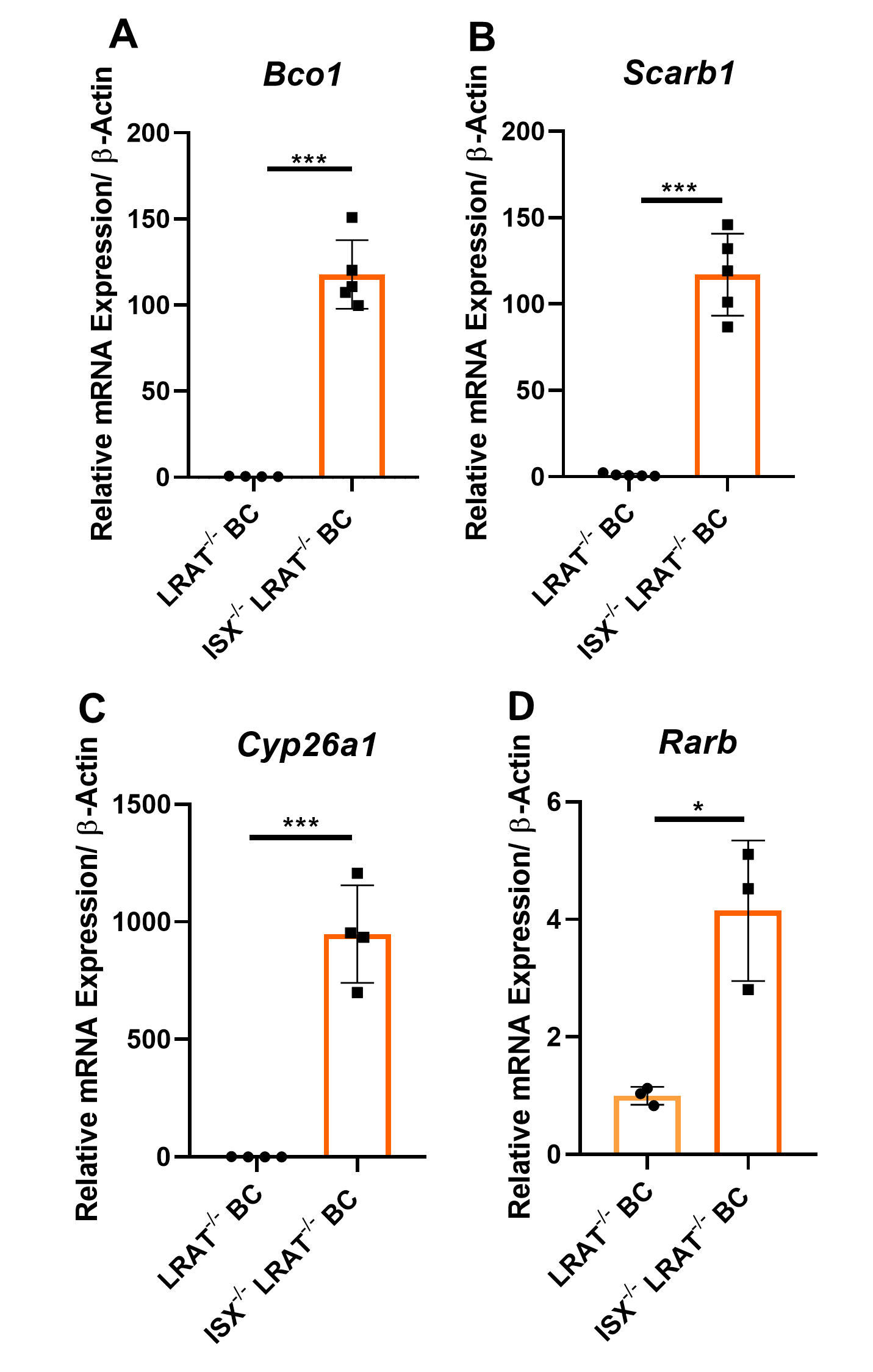
**

**Supplementary Figure 3. Genetic deletion of *Isx* gene restores Bco1 expression in the LRAT-deficient intestine**. *Lrat^-/-^* and *Lrat^-/-^/Isx^-/-^* (DKO) (n= 4-5 genotype) were fed with β-carotene (BC) diet for 4 weeks. A,B, qRT-PCR analysis of *Scarb1* and *Bco1* mRNA levels in total RNA preparation of the jejunum. C,D qRT-PCR analysis of *Cyp26a1* and *Rarb* mRNA levels in total RNA preparation of the liver. Values were normalized to the mRNA levels of *Lrat^-/-^* and are displayed as mean ±SD. *, p < 0.05; ***p < 0.0001 using unpaired two tail *t* test.
